# The structure, function and properties of sirohaem decarboxylase - an enzyme with structural homology to a transcription factor family that is part of the alternative haem biosynthesis pathway

**DOI:** 10.1111/mmi.12656

**Published:** 2014-06-18

**Authors:** David J Palmer, Susanne Schroeder, Andrew D Lawrence, Evelyne Deery, Susana A Lobo, Ligia M Saraiva, Kirsty J McLean, Andrew W Munro, Stuart J Ferguson, Richard W Pickersgill, David G Brown, Martin J Warren

**Affiliations:** 1 School of Biosciences, University of KentGiles Lane, Canterbury, Kent, CT2 7NJ, UK; 2 Instituto de Tecnologia Química e Biológica António Xavier, Universidade Nova de LisboaAv. da República, 2780-157, Oeiras, Portugal; 3 Manchester Institute of Biotechnology, Faculty of Life Sciences, University of Manchester131 Princess Street, Manchester, M1 7DN, UK; 4 Department of Biochemistry, University of OxfordSouth Parks Road, Oxfordshire, OX1 3QU, UK; 5 School of Biological and Chemical Sciences, Queen Mary University of LondonMile End Road, London, E1 4NS, UK

## Abstract

Some bacteria and archaea synthesize haem by an alternative pathway, which involves the sequestration of sirohaem as a metabolic intermediate rather than as a prosthetic group. Along this pathway the two acetic acid side-chains attached to C12 and C18 are decarboxylated by sirohaem decarboxylase, a heterodimeric enzyme composed of AhbA and AhbB, to give didecarboxysirohaem. Further modifications catalysed by two related radical SAM enzymes, AhbC and AhbD, transform didecarboxysirohaem into Fe-coproporphyrin III and haem respectively. The characterization of sirohaem decarboxylase is reported in molecular detail. Recombinant versions of *Desulfovibrio desulfuricans*, *Desulfovibrio vulgaris* and *Methanosarcina barkeri* AhbA/B have been produced and their physical properties compared. The *D. vulgaris* and *M. barkeri* enzyme complexes both copurify with haem, whose redox state influences the activity of the latter. The kinetic parameters of the *D. desulfuricans* enzyme have been determined, the enzyme crystallized and its structure has been elucidated. The topology of the enzyme reveals that it shares a structural similarity to the AsnC/Lrp family of transcription factors. The active site is formed in the cavity between the two subunits and a AhbA/B-product complex with didecarboxysirohaem has been obtained. A mechanism for the decarboxylation of the kinetically stable carboxyl groups is proposed.

## Introduction

**Fig. 1:**
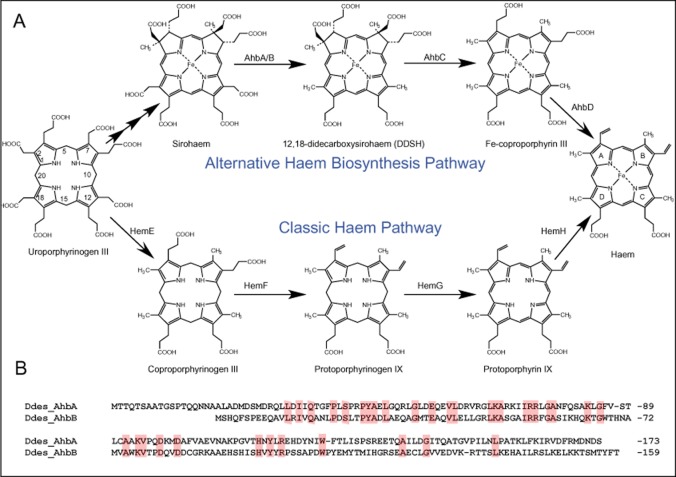
Comparison of the ‘alternative’ and ‘classic’ pathways for the biosynthesis of haem.A. Direct comparison of the two pathways showing the transformation of uroporphyrinogen III into haem via the ‘classic’ and ‘alternative’ routes with the relevant intermediates. The numbering of the tetrapyrrole macrocycle is highlighted in uroporphyrinogen and the labelling of the 4 pyrrole-derived rings (A-D) is shown in haem.B. Comparison of the sequences of *D. desulfuricans* AhbA and AhbB, which constitute the sirohaem decarboxylase. Conserved residues are highlighted.

The modified tetrapyrroles such as haems, chlorophylls, sirohaem, haem *d*_1_, cobalamins (vitamin B_12_) and coenzyme F_430_, are characterized by a large macrocyclic ring into which is chelated a central metal ion (Warren and Scott, 1990). They play important roles in many essential life processes and are recognized by their striking chromophores, allowing them to be dubbed as the pigments of life (Battersby, 2000). Haem is the best recognized member of this molecular fraternity and is also the most diverse in terms of the function the prosthetic group plays in biological systems. It is a component of many enzymes, cytochromes, sensors, regulators and transport systems and is found in all kingdoms of life (Hamza and Dailey, 2012). The biosynthesis of haem is also well understood in molecular detail, being derived from the common tetrapyrrole primogenitor uroporphyrinogen III in four steps along the classic pathway (Fig. 1). This pathway, which is found in eukaryotes and most eubacteria, involves the decarboxylation of the four acetate side-chains of uroporphyrinogen III to yield coproporphyrinogen III, two oxidative decarboxylations of the northern propionic acid side-chains to give protoporphyrinogen IX, the removal of six protons and electrons to generate protoporphyrin IX and finally iron chelation to produce haem (Hamza and Dailey, 2012).

In contrast, a new branch of tetrapyrrole biosynthesis has been recently described that is responsible for an alternative haem biogenesis pathway that also accounts for the synthesis of haem *d*_1_ (Bali *et al*., 2011; 2014a,b). This route employs sirohaem, the prosthetic group of sulphite and nitrite reductases, as an intermediate (Fig. 1). Here, sirohaem undergoes decarboxylation of the southern acetic acid side-chains, attached to C12 (ring C) and C18 (ring D) of the macrocycle, to give didecarboxysirohaem in a reaction catalysed by sirohaem decarboxylase, which is composed of two subunits called AhbA and AhbB (Bali *et al*., 2011). Subsequently, two S-adenosyl-L-methionine-dependent enzymes, AhbC and AhbD, then oversee the transformation of didecarboxysirohaem into Fe-coproporphyrin and haem respectively (Bali *et al*., 2011). The classic and alternative pathways for haem synthesis are outlined in Fig. 1.

Confirmation that AhbA/B is a sirohaem decarboxylase came from studies with extracts of recombinant *Escherichia coli* overproducing the two protein subunits from either *Desulfovibrio desulfuricans* or *Desulfovibrio vulgaris* as incubation of the extracts with sirohaem gave rise to the quantitative production of didecarboxysirohaem by the removal of the carboxylate groups of the acetic acid side-chains (Bali *et al*., 2011). The two subunits share a degree of similarity to each other (Fig. 1) as well as to the NirD, L, G and H enzymes that are associated with haem *d*_1_ synthesis (Bali *et al*., 2011). All of these proteins share significant sequence similarity with the Lrp/AsnC family of transcription factors. Indeed, initially, it was thought that the role of these proteins was to act as transcription regulators (Xiong *et al*., 2007).

The *Methanosarcina barkeri* AhbA/B proteins have also been overproduced in *E. coli* and, when purified, were shown to form a heterodimeric complex (Kuhner *et al*., 2014). Moreover, the proteins were found to bind haem, suggesting that the enzyme may be sensitive to feedback inhibition. However, no functional studies were performed on the purified enzyme and it is not clear if AhbA or AhbB, by themselves, are active as decarboxylases.

The two decarboxylation reactions catalysed by AhbA/B are reminiscent of the decarboxylations of the four acetate side-chains mediated by uroporphyrinogen decarboxylase from the classic haem pathway (Whitby *et al*., 1998; Phillips *et al*., 2003). Unusually for a decarboxylation reaction the uroporphyrinogen decarboxylase does not require any cofactor or metal ion, but instead uses the nitrogen within the individual pyrrole rings of the substrate as electron sinks. Nonetheless, uroporphyrinogen decarboxylases are highly proficient enzymes as the spontaneous rate of decarboxylation of a substrate model compound was found to have a half-life in excess of two billion years (Lewis and Wolfenden, 2008). To enhance the reaction the enzyme provides a protonated basic residue, Arg 37 in human uroporphyrinogen decarboxylase, to assist the carboxylate removal (Silva *et al*., 2010).

Herein, we report the biochemical characterization of a number of different AhbA/B enzyme systems, including *D. desulfuricans*, *D. vulgaris* and *M. barkeri*. We reveal that a number of these protein complexes bind haem and demonstrate that in one case the haem acts as a redox control element. We also present the crystal structures of AhbA/B from *D. desulfuricans*, as both an apo heterodimeric structure and as a product bound complex. A reaction mechanism is proposed based on the presence of a number of conserved amino acid residues.

## Results and discussion

### AhbA and AhbB form a heteromeric complex

**Fig. 2:**
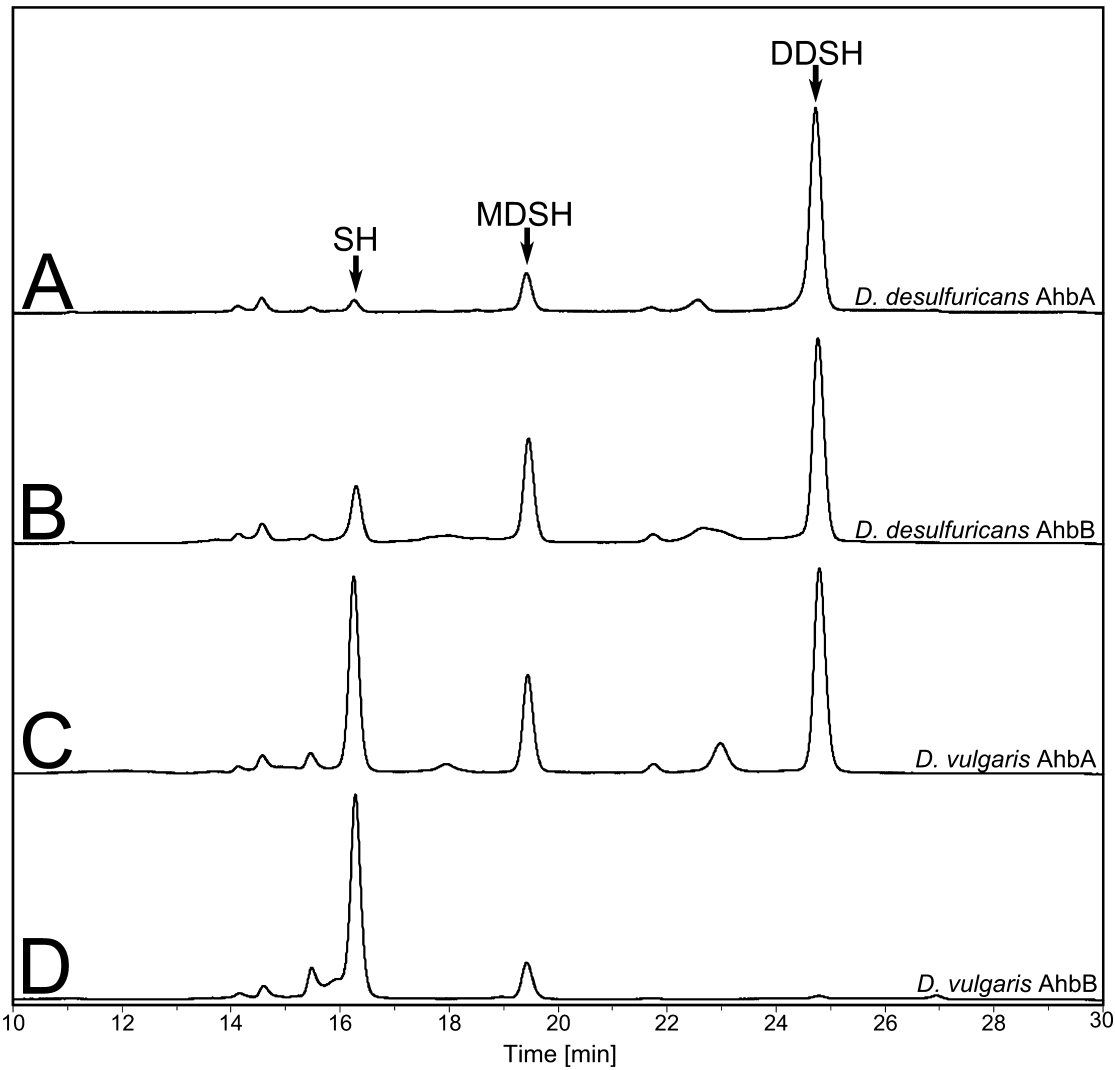
Reaction profile of sirohaem decarboxylases. HPLC traces, recorded at a wavelength of 380 nm, after incubation of sirohaem with excess purified (A) *D. desulfuricans* AhbA, (B) *D. desulfuricans* AhbB, (C) *D. vulgaris* AhbA, and (D) *D. vulgaris* AhbB. The arrows indicate the peaks relating to sirohaem (SH, ∼ 16 min), monodecarboxysirohaem (MDSH, ∼ 19.5 min) and didecarboxysirohaem (DDSH, ∼ 25 min).

Although it has been shown that together AhbA and AhbB are able to decarboxylate sirohaem to didecarboxysirohaem (Bali *et al*., 2011) the individual action of each protein was not characterized. To address the activity of each protein, *ahbA* and *ahbB* from *D. desulfuricans* and *D. vulgaris* were cloned into distinct plasmids and expressed separately in *E. coli*. When expressed individually the encoded proteins were found to be highly unstable and could only be purified at very low concentrations due to their tendency to precipitate. Incubations of individual AhbA and AhbB proteins in excess with sirohaem yielded mixtures with varying levels of sirohaem, monodecarboxysirohaem and didecarboxysirohaem (Fig. 2). The monodecarboxysirohaem was not characterized further and hence it was not determined as to whether it was due to loss of the carboxylic acid from the C12 or C18 side-chain. However, the result demonstrates that the individual subunits, AhbA and AhbB, are capable of decarboxylating sirohaem by themselves. It is possible that the individual subunits form homodimers, which likely represents the active species.

**Fig. 3:**
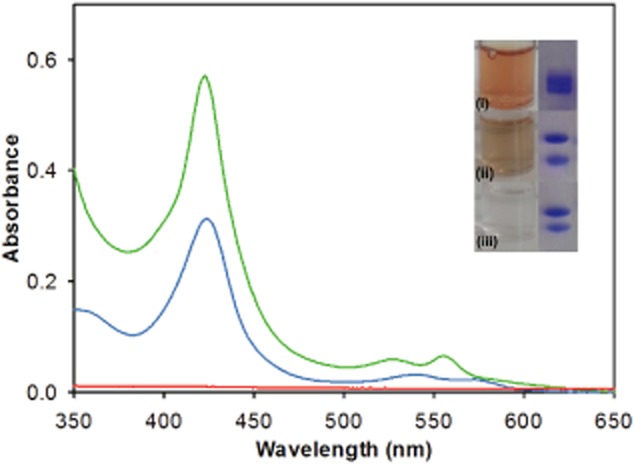
UV-visible spectra of purified sirohaem decarboxylase. The spectra of *M. barkeri* AhbA/B (green), *D. vulgaris* AhbA/B (blue), and *D. desulfuricans* AhbA/B (red) are shown. Inset: SDS gel and picture of solutions of AhbA/B proteins purified from *E. coli* strains overproducing (i) *M. barkeri* AhbA/B, (ii) *D. vulgaris* (haem-loaded) AhbA/B, and (iii) *D. desulfuricans* AhbA/B.

It was therefore assumed that both AhbA and AhbB have a stabilizing effect on each other by forming a complex. Hence, both genes from *D. desulfuricans* were cloned into a single vector (with *ahbA* being untagged and *ahbB* encoding an N-terminal hexa-histidine tag) and subsequently coexpressed in *E. coli*. Purification of protein from *E. coli* expressing this construct resulted in a high yield of stable protein, and SDS analysis showed that AhbA and AhbB had copurified together (Fig. 3), demonstrating that these proteins form a heteromeric complex. Gel filtration studies revealed that the AhbA/B complex from *D. desulfuricans* form a single heterodimeric species with a mass of 40 kDa, which correlates closely with the expected mass of 39 kDa (Fig. S1). It has been reported previously that, in most archaea harbouring these genes, *ahbA* and *ahbB* are found as a fusion (Storbeck *et al*., 2010), which may explain why the enzymes are unstable when produced individually.

To test this hypothesis further *ahbA* and *ahbB* from the other sulphate reducer, *D. vulgaris*, were expressed and the encoded proteins purified in an identical manner. Again copurification yielded much higher quantities of stable protein in comparison to the isolation of individual proteins. Surprisingly, gel filtration of the AhbA/B complex from *D. vulgaris* revealed the presence of not only a dimeric complex but also a tetrameric complex with masses of 44 and 94 kDa respectively (close to the estimated masses of 40 and 81 kDa, which includes the histidine tag). Interestingly, the *D. vulgaris* protein purified with a light brown colour in contrast to the colourless isolated *D. desulfuricans* protein complex.

UV-Vis analysis of the *D. vulgaris* AhbA/B complex revealed an absorption peak at 420 nm, consistent with the presence of either an Fe-S complex or a haem group. However, analysis of the protein sequences showed no CXXXCXXC motif which is conserved in many iron sulphur centre containing proteins (Sofia *et al*., 2001). It was, therefore, postulated that the absorption at 420 nm may be due to a bound haem group in low occupancy. To investigate this possibility further 6 mM haemin was added to the resuspended cell pellet of the strain overproducing the *D. vulgaris* AhbA/B prior to sonication in an attempt to load the complex with the prosthetic group. This approach did indeed result in purified protein with greater brown/orange colouration. Haem-loaded *D. vulgaris* AhbA/B had a Soret band at 423 nm and *αβ* bands at 555 and 527 nm (reduced spectrum) (Fig. 3). The haem was retained with the protein during gel filtration and did not affect the oligomeric state of the complex. It has recently been reported that *M. barkeri* AhbA/B also copurifies with a tightly bound haem (Kuhner *et al*., 2014). We, also, cloned the *M. barkeri ahbA/B* genes and produced recombinant enzyme in *E. coli*. The purified *M. barkeri* AhbA/B complex has a higher haem occupancy than the *D. vulgaris* AhbA/B. The absorption bands in the UV-Vis spectrum of haem-bound *D. vulgaris* AhbA/B is slightly shifted in comparison to the *M. barkeri* AhbA/B (426, 530 and 559 nm) (Kuhner *et al*., 2014).

Further confirmation for the presence of haem was obtained from pyridine haemochrome assays (Berry and Trumpower, 1987), which were performed with both the exogenous haem-loaded *D. vulgaris* AhbA/B and *M. barkeri* AhbA/B. Difference spectra of reduced minus oxidized absorption of the pyridine haemochrome from *M. barkeri* displayed a peak at 556 nm (Fig. S2), which corresponds to a *b* type haem. The haem-loaded complex from *D. vulgaris* again showed a slightly shifted spectrum with a haemochrome peak at 554 nm, shorter than that reported for haem *b*, but longer than that of a haem *c* (∼ 550 nm) (Fig. S2).

Low levels of haem could be removed from the haem-loaded *D. vulgaris* AhbA/B complex using haem extraction methods (Lubben and Morand, 1994). The presence of the extracted haem was confirmed by mass spectrometry, but the majority remained bound in the protein pellet. However, haem could not be detected on an SDS gel using a haem staining protocol; this suggests that it is likely to be a haem *b* bound to *D. vulgaris* AhbA/B. The tight binding of haem to the enzyme complex may explain the shift observed in the UV-Vis spectra of the proteins and their pyridine haemochrome derivatives. In contrast, it was much easier to remove the haem from the *M. barkeri* AhbA/B complex.

### *D. desulfuricans* AhbA/B kinetics

The decarboxylations of sirohaem at C12 and C18 have little impact on the conjugation of the tetrapyrrole ring system and therefore the UV-Vis spectra of sirohaem, monodecarboxysirohaem and didecarboxysirohaem are practically identical. Also, given the oxygen sensitivity of the substrates and products the only way to measure sirohaem decarboxylase activity has been through the use of a stopped assay under anaerobic conditions followed by quantification with mass spectrometry. Despite these technical challenges the kinetic parameters for the sirohaem decarboxylase were investigated for the *D. desulfuricans* AhbA/B.

**Fig. 4:**
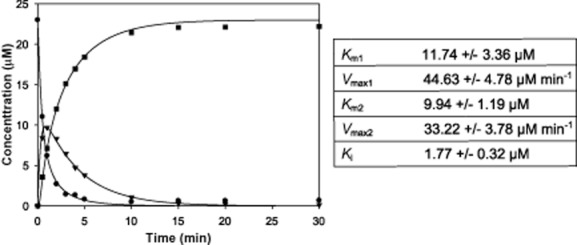
Kinetic analysis sirohaem decarboxylase. Data from the reaction of 23 μM sirohaem with 2.3 μM *D. desulfuricans* AhbA/B. Initially, sirohaem (circles) is decarboxylated to monodecarboxysirohaem (triangles), which is in turn decarboxylated to didecarboxysirohaem (squares). These reactions follow a competitive inhibition model for both substrates and the product. Data were fitted using in-house software.

The assays revealed the rapid production of monodecarboxysirohaem, which is then gradually converted to didecarboxysirohaem (Fig. 4). These results support a single active site with a competitive inhibition model for the two substrates (sirohaem and monodecarboxysirohaem). To achieve a good fit for the data a further inhibitory constant is added due to the tight binding nature of the product (as discussed later).

The rate equations used are as follows:


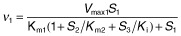



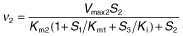


where *ν*_1_ is rate of sirohaem decarboxylation, *S*_1_ is the concentration of sirohaem, *ν*_2_ is the rate of monodecarboxysirohaem decarboxylation, *S*_2_ is the concentration of monodecarboxysirohaem, *S*_3_ is the concentration of didecarboxysirohaem, *K*_i_ is the tight-binding constant of didecarboxysirohaem to the enzyme and *V*_max1_/*V*_max2_ and *K*_m1_/*K*_m2_ are the kinetic parameters of each reaction respectively.

Data were averaged from three replicates and fitted using in-house software. *K*_m1_ = 11.74 ± 3.36 μM, *V*_max1_ = 44.63 ± 4.78 μM min^−1^, *K*_m2_ = 9.94 ± 1.19 μM, *V*_max2_ = 33.22 ± 3.78 μM min^−1^, *K*_i_ = 1.77 ± 0.32 μM.

These data show that both reactions have very similar parameters. Although the initial reaction has a higher *V*_max_, this is compensated for by the intermediate having a slightly increased affinity for the enzyme, which prevents the build-up of monodecarboxysirohaem. The binding constant for the product is significantly lower than that of the substrates, explaining the tight binding of the product to the enzyme.

### Haem oxidation state regulates *M. barkeri* AhbA/B activity

**Fig. 5:**
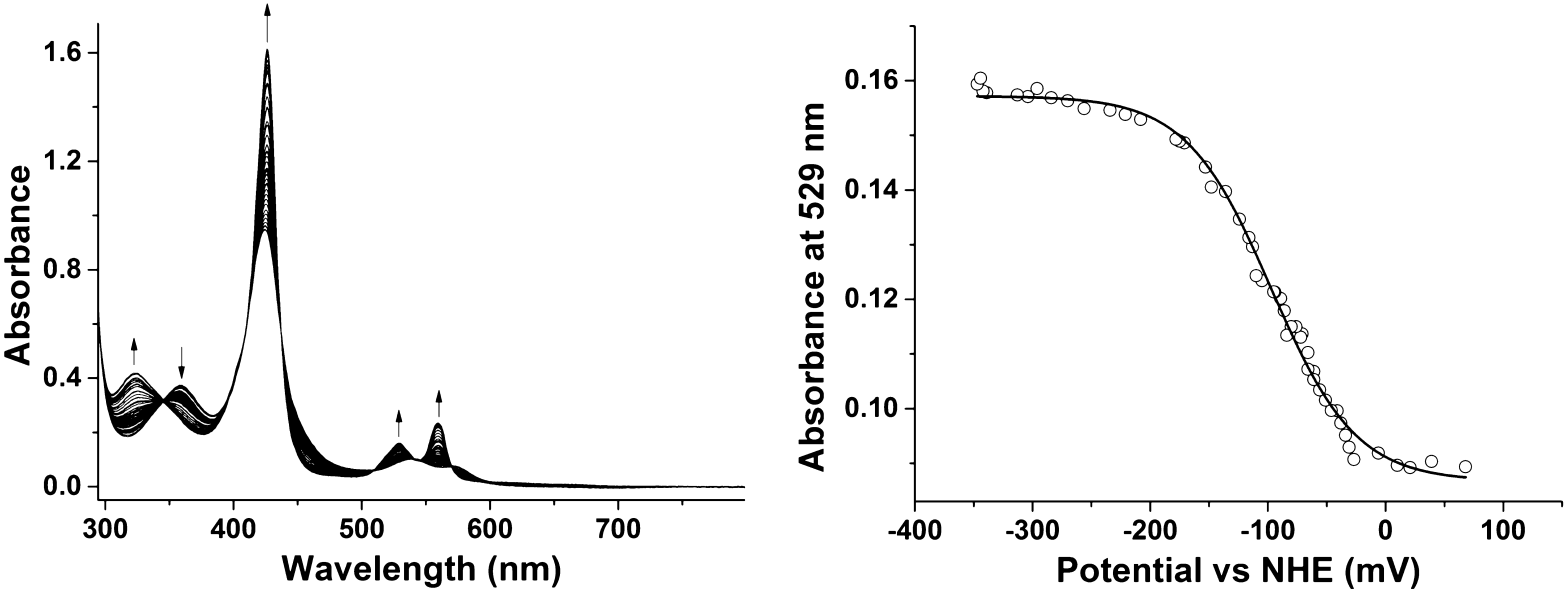
Redox titration of *M. barkeri* AhbA/B complex.A. UV-Vis spectra recorded during the redox titration. The arrows show directional changes of peaks as a reductive titration progresses.B. Plot of absorbance at 529 nm against redox potential versus normal hydrogen electrode (NHE). The redox potential for the Fe^3+^/Fe^2+^ couple of the bound haem iron is −98 ± 3 mV.

The activity of the purified AhbA/B enzymes from *D. vulgaris*, *D. desulfuricans* and *M. barkeri* were compared. Both *D. vulgaris* and *D. desulfuricans* AhbA/B complexes display quantitative conversion of sirohaem to didecarboxysirohaem following incubation at 37°C for 15 h. However, similar incubations of *M. barkeri* AhbA/B with sirohaem show low turnover (Fig. S3). Repeating the assay in the presence of sodium dithionite enhances the activity to the protein, indicating that the haem bound to the AhbA/B complex must be reduced in order for the enzyme to be most active. Treatment with excess dithionite results in a 6.5-fold increase in the activity of the enzyme. To investigate this further the reaction was repeated with AhbA/B protein that had been oxidized by exposure to potassium ferricyanide prior to buffer exchange. Again low activity was observed with the oxidized protein, which could be recovered by the addition of sodium dithionite (Fig. S3). A redox titration of the haem bound to the *M. barkeri* AhbA/B complex gave a midpoint redox potential of −98 ± 3 mV from Q-band data at 529 nm (Fig. 5). Gel filtration of *M. barkeri* AhbA/B under reducing conditions showed no change in oligomerization state.

We have therefore demonstrated that the redox state of the haem *b* in the *M. barkeri* AhbA/B modulates the activity of the enzyme. Oxidation of the haem may cause a conformational change in the complex, thereby reducing activity. It has been speculated that the haem acts on the AhbA/B complex as a feedback inhibitor (Kuhner *et al*., 2014), but this is clearly not the case as the enzyme is active with the prosthetic group bound. Interestingly the haem-loaded AhbA/B complex from *D. vulgaris* showed no difference in activity in the presence or absence of a reducing agent. Similarly, reducing agents did not affect the activity of *D. desulfuricans* AhbA/B (data not shown). Thus haem would appear to modulate activity in some, but not all, of these enzymes and likely reflects a regulatory mechanism for the pathway.

### Product binding of complexes

It has recently been demonstrated that some enzymes from the aerobic cobalamin biosynthesis pathway tightly bind their products, protecting the unstable intermediates from the external environment (Deery *et al*., 2012). Such a process is an inherent property of enzymes involved in direct metabolite channelling, whereby the product of the first enzyme is only released to the subsequent enzyme in a pathway when the two enzymes are in close proximity. Given the oxygen-sensitive nature of sirohaem and didecarboxysirohaem, we investigated whether the AhbA/B enzymes are also able to bind their products tightly. Constructs containing *cysG*, which encodes the sirohaem synthase from *E. coli* (Warren *et al*., 1990) (Fig. S4), and *ahbA/B* from each organism were generated in order to assess the product binding capacity of the AhbA/B complexes.

When grown in the presence of excess CysG both the *D. vulgaris* and *D. desulfuricans* AhbA/B complexes copurified with purple coloured compounds, consistent with the binding of a substrate or product. HPLC-MS analysis showed that both AhbA/B complexes copurified largely with didecarboxysirohaem, although a small amount of monodecarboxysirohaem (approximately 10%) was also detected. The AhbA/B complex from *M. barkeri* does not bind any substrate or product. The tight binding of didecarboxysirohaem by the AhbA/B complexes from *D. vulgaris* and *D. desulfuricans* may be related to the need to protect the unstable product from the intracellular environment. Didecarboxysirohaem may be handed on to the next enzyme in the pathway, AhbC, by substrate channelling. It is possible that this does not occur in *M. barkeri* as the cell may be able to allow free didecarboxysirohaem within the cellular environment, possibly because it grows in even more anaerobic environments.

### Purification of novel AhbA/B chimeric complexes

There is a high degree of sequence similarity between AhbAs and AhbBs from different species. *D. vulgaris* AhbA shares 72% identity with that of *D. desulfuricans* and 41% with *M. barkeri*, and *D. desulfuricans* AhbA shares 40% identity with *M. barkeri* AhbA. Likewise, *D. vulgaris* AhbB shares 67% identity with AhbB from *D. desulfuricans* and 37% with *M. barkeri* AhbB, and *D. desulfuricans* and *M. barkeri* AhbBs share 35% identity. We therefore investigated whether it is possible to form chimeric complexes with subunits from different species. Such complexes may highlight specific attributes of one of the subunits, such as haem or substrate binding capabilities. To observe if AhbA and AhbB proteins from separate organisms could interact with each other, constructs were made containing different sets of *ahbA* and *ahbB* genes.

*D. vulgaris* AhbA and *D. desulfuricans* AhbB could be copurified as a complex and exhibited a similar light brown colouration to the *D. vulgaris* AhbA/B. Both dimeric and tetrameric complexes were observed during gel filtration with peaks at 41 kDa and 97 kDa (Fig. S5). This complex showed full enzymatic activity when incubated with purified sirohaem (Fig. S6). *D. desulfuricans* AhbA was also found to copurify with the *D. vulgaris* AhbB. This complex was colourless and purified only as a dimer running at a mass of 41 kDa (Fig. S5). The *in vitro* activity assay showed that this complex is also fully active (Fig. S6). From these results it would seem that AhbA from *D. vulgaris* is involved with the formation of AhbA/B tetramers. The protein also conveys a greater ability to bind haem within the cells prior to purification. Mixing the AhbA and AhbB from the *Desulfovibrio* species with those from *M. barkeri* did not realize any soluble protein.

### Overall structure of AhbA/B

**Fig. 6:**
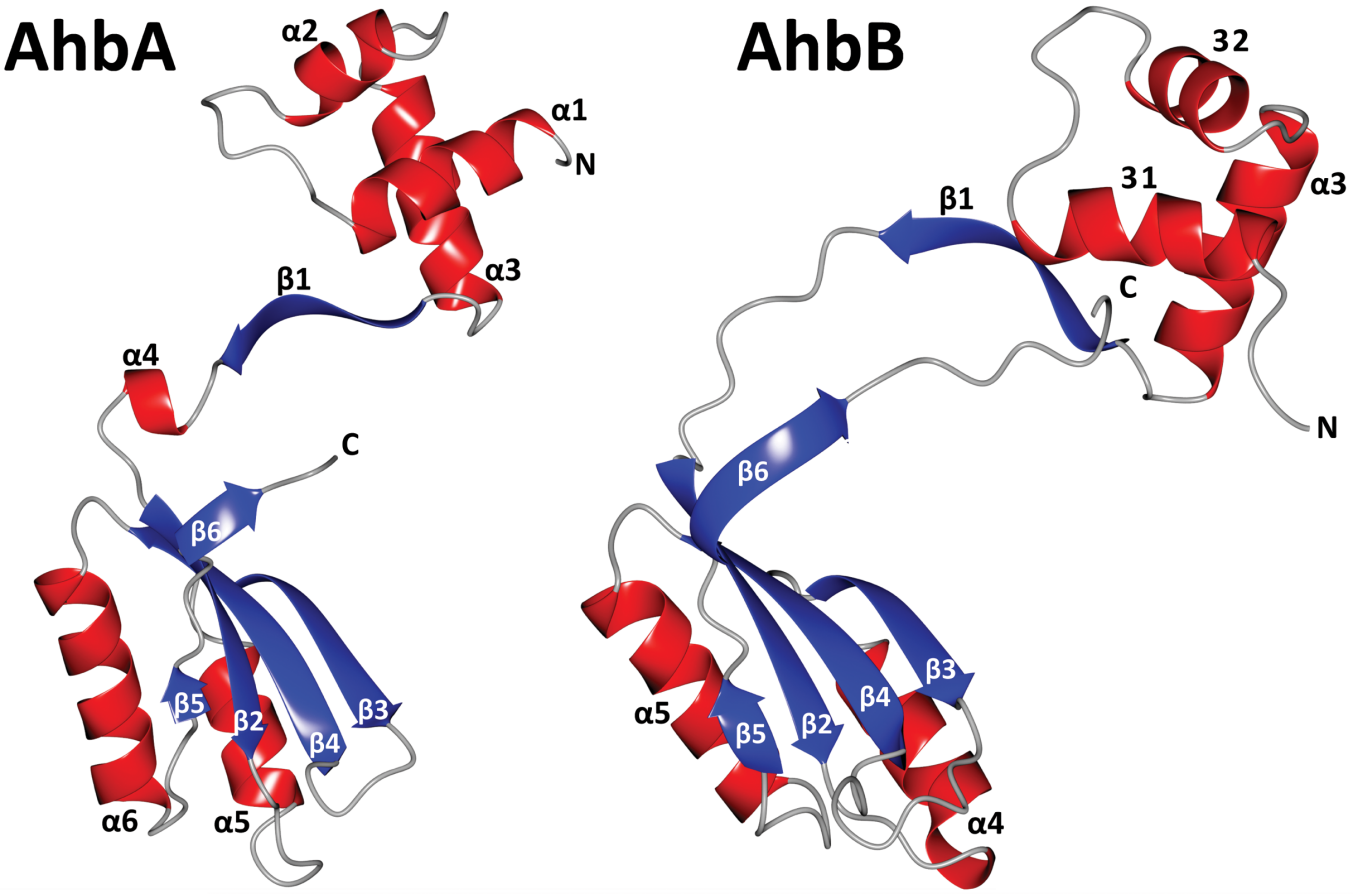
The structures of the individual subunits of the sirohaem decarboxylase. The monomers of AhbA (left) and AhbB (right,) are coloured by secondary structural elements, (helices = red, sheets = blue) and labelled by secondary structure. The termini are indicated N and C. Structural diagrams were produced using CCP4 molecular graphics program (McNicholas *et al*., 2011).

Crystallography was attempted with all three homologues of the AhbA/B complex, but only the *D. desulfuricans* form produced crystals amenable to structure determination. Native crystals diffracted to 2.23 Å and the structure was solved using 3 wavelength SeMet MAD data. The initial structure confirmed the formation of a heterodimer of AhbA and AhbB with two heterodimers in the asymmetric unit (Fig. 6). The full sequence of AhbB was correctly traced in the structure except for the histidine tag, which was disordered. The majority of AhbA was also identified with only the first 20 and last 10 amino acids in disordered regions. A summary of the crystallographic data statistics can be found in Table 1.

**Table 1:** Summary of crystallographic data and refinement statistics

	Native AhbA/B	Siroheme soaked AhbA/B	SeMet AhbA/B (peak)	SeMet AhbA/B (inflection)	SeMet AhbA/B (remote high)
Data collection					
Resolution (Å)	69.8–2.23 (2.29)	44–1.97 (2.02)	75.3–2.64 (2.71)	78.26–2.65 (2.72)	78.32–2.71 (2.78)
Wavelength (Å)	0.92	0.98	0.97917	0.97935	0.97854
Space group	P2_1_2_1_2_1_	P2_1_2_1_2_1_	P2_1_2_1_2_1_	P2_1_2_1_2_1_	P2_1_2_1_2_1_
Cell constants (Å)	55.85 × 78.62 × 151.4	55.3 × 78.6 × 150.3	55.63 × 78.22 × 150.6	55.62 × 78.26 × 150.7	55.64 × 78.32 × 150.7
Multiplicity	5.0 (3.8)	5.3 (4.9)	12.7 (9.9)	12.8 (10.1)	13.0 (11.9)
Mean (I)/SD(I)	13.1 (2.0)	13.38 (2.5)	20.4 (3.0)	20.9 (3.0)	21.3 (3.7)
Observations	160 189 (8051)	245 471 (16 175)	254 097 (13 624)	252 537 (13 697)	24 924 (15 998)
Unique observations	32 358 (2121)	46 648 (3309)	19 969 (1383)	19 771 (1356)	18 595 (1340)
*R*_merge_	4.6 (69.3)	8.2 (80.4)	8.9 (70.7)	8.7 (69.6)	8.9 (66)
Completeness (%)	97.6 (89.6)	99.2 (97.0)	99.6 (95.1)	99.6 (95.3)	99.9 (99.3)
Ligand occupancy (%)	–	60	–	–	–
Refinement					
*R*_factor_ (%)	19.8	20.4	–	–	–
*R*_free_ (%)	23.6	26.6	–	–	–
r.m.s.d.					
Bond length (Å)	0.0156	0.017	–	–	–
Bond angles (°)	1.6	2.2	–	–	–
No. of residues	704	704	–	–	–
No. of waters	252	576	–	–	–

Data resolution cut at CC1/2 0.5. Native and selenomethionine data processed using XDS in xia2, substrate soaked data processed using XDS.

The AhbA/B complex is a heterodimer formed from two similar protein subunits (Fig. 1) that share a near identical fold (Fig. 6). Both proteins consist of an N-terminal and a C-terminal domain joined by a linking region. The N-terminal domain contains 3 helices (α1–α3), two of which (α2 and α3) form a helix–turn–helix (HTH) motif. Helix α1 interacts with this motif via a hydrophobic core. The N-terminal domain then continues with a β-strand (β1) before the linking region joins the two domains. The AhbA linker contains a single turn helix (α4) that is not observed in AhbB. This AhbA α4-helix is stabilized by the N-terminal methionine of AhbB. The linker region for AhbB consists of a longer loop region the flexibility of which is inferred from the poorer electron density observed.

The C-terminal domains of each protein are reminiscent of the previously described RAM (regulation of amino acid metabolism) domain with a βαββαβ fold, and the closely related ‘ACT domain’ (Chipman and Shaanan, 2001; Ettema *et al*., 2002). However, as there is no evidence that AhbA/B is related to amino acid metabolism it will be subsequently referred to as a ‘RAM-like’ domain. A four-stranded β sheet is formed from β2-5 with 2 helices packed against one face (AhbA α5 and α6, AhbB α4 and α5) stabilizing the β-sheets through hydrophobic interactions. In AhbA α5 interacts with β3 and β4, and α6 with β2. Similarly, in AhbB α4 interacts with β3 and β4, and α5 interacts with β2 and β4. After a short loop, one final strand (β6) is formed at the C-terminal end of each monomer.

**Fig. 7:**
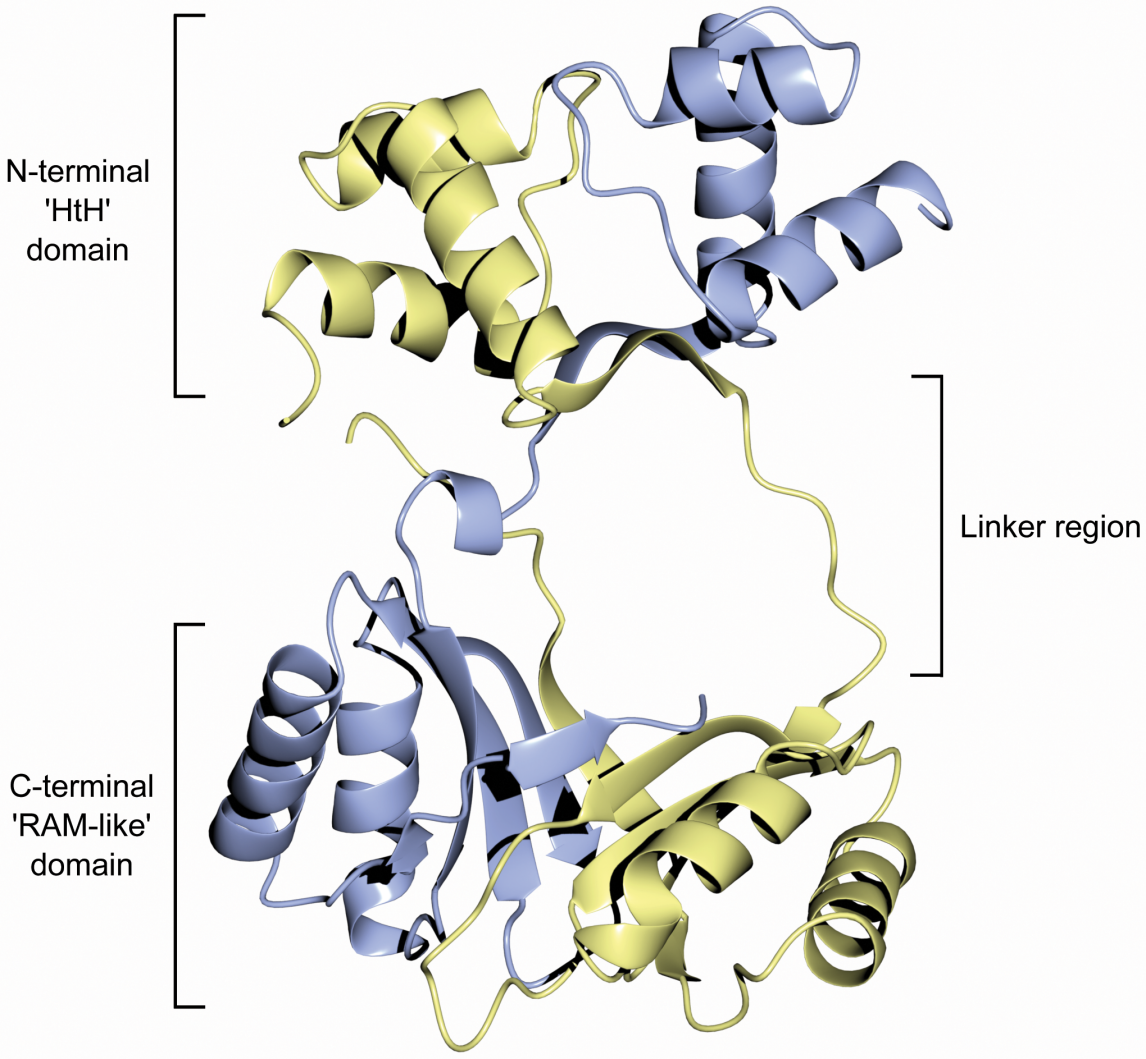
The structure of the AhbA/B heterodimer from *D. desulfuricans*. The complex is formed of AhbA (blue) and AhbB (yellow). Coordinates and structure factor files were submitted in the protein databank with accession code 4CZD. Structural diagrams were produced using CCP4 molecular graphics program (McNicholas *et al*., 2011).

The Ahb monomers form a closely associated complex (Fig. 7). At the base of the N-terminal domain, β1 from each protein forms an anti-parallel β-ribbon by direct hydrogen bonding at the start of the linking region. At the C-terminus the two RAM-like domains dimerize via hydrophobic interactions at the base of each domain forming a sandwich of β-sheets with a hydrophobic cleft (Fig. 7). Finally, the C-terminal β-strand (β6) directly hydrogen bonds to β3 of the opposing monomer to form a tight interaction. The effect of the different linkers between the two domains that form the heterodimer AhbA/B is that the central cavity formed between the N- and C-terminal domains is opened up to form the substrate-binding cavity (see below for more details on substrate binding).

### Comparison to AsnC/Lrp DNA binding proteins

As mentioned previously the AhbA and AhbB proteins were both annotated as members of the AsnC transcriptional regulator family due to sequence homology, prior to the discovery that they were in fact involved in alternative haem synthesis (Bali *et al*., 2011). It is interesting to note that despite their catalytic role they have an identical 3D structural arrangement as members of the Lrp/AsnC family of transcriptional regulators, which similarly contain an N-terminal HTH motif and a C-terminal RAM domain. Superimposing *D. desulfuricans* AhbA onto a monomer of *E. coli* AsnC (pdb 2CG4) gives a poorer structural alignment [root mean squared deviation (r.m.s.d.) 3.9 Å for 123 Cα atoms] than aligning AhbB (r.m.s.d. 2.7 Å for 140 Cα atoms). These values demonstrate that AhbA/B and *E. coli* AsnC have a high degree of structural homology. Superimposing the entire AhbA/B dimer onto AsnC using AhbB as a template demonstrates that the N-terminal domain of AhbA is angled towards that of AhbB. This movement, combined with the flexible linker of AhbB, effectively opens up one side of the dimer, allowing access to the cleft formed by the C-terminal domains. *E. coli* AsnC and other DNA binding Lrp/AsnC proteins show a much more condensed symmetrical structure.

The structural homology of AhbA/B to *E. coli* AsnC suggests that they may still function as transcriptional regulators as well as enzymes. Similarly, a homologue of AhbA and AhbB, involved in the haem *d*_1_ biosynthesis pathway, NirL, from *Heliophilum fasciatum*, has been shown to bind DNA; specifically to the putative promoter of *nirJ2*, which encodes a gene homologous to AhbC and AhbD (Xiong *et al*., 2007). The movement in the DNA binding region of AhbA, in comparison to AsnC, may mean that the HTH motifs are not in the optimal configuration to bind DNA. Lrp-like proteins have been noted to bind a variety of amino acids which effect DNA affinity, DNA bending, and altering and stabilizing tertiary and quaternary protein structure (Calvo and Matthews, 1994; Madhusudhan *et al*., 1997; Chen *et al*., 2001; Brinkman *et al*., 2002; Thaw *et al*., 2006; Ren *et al*., 2007). Therefore, it is possible that effector binding could induce conformational changes to allow repositioning of AhbA, or induce multimerization of Lrp/AsnC proteins, which may represent a possible DNA binding state for AhbA/Bs (Jeong *et al*., 2013).

### AhbA/B active site and mechanism of action

**Fig. 8:**
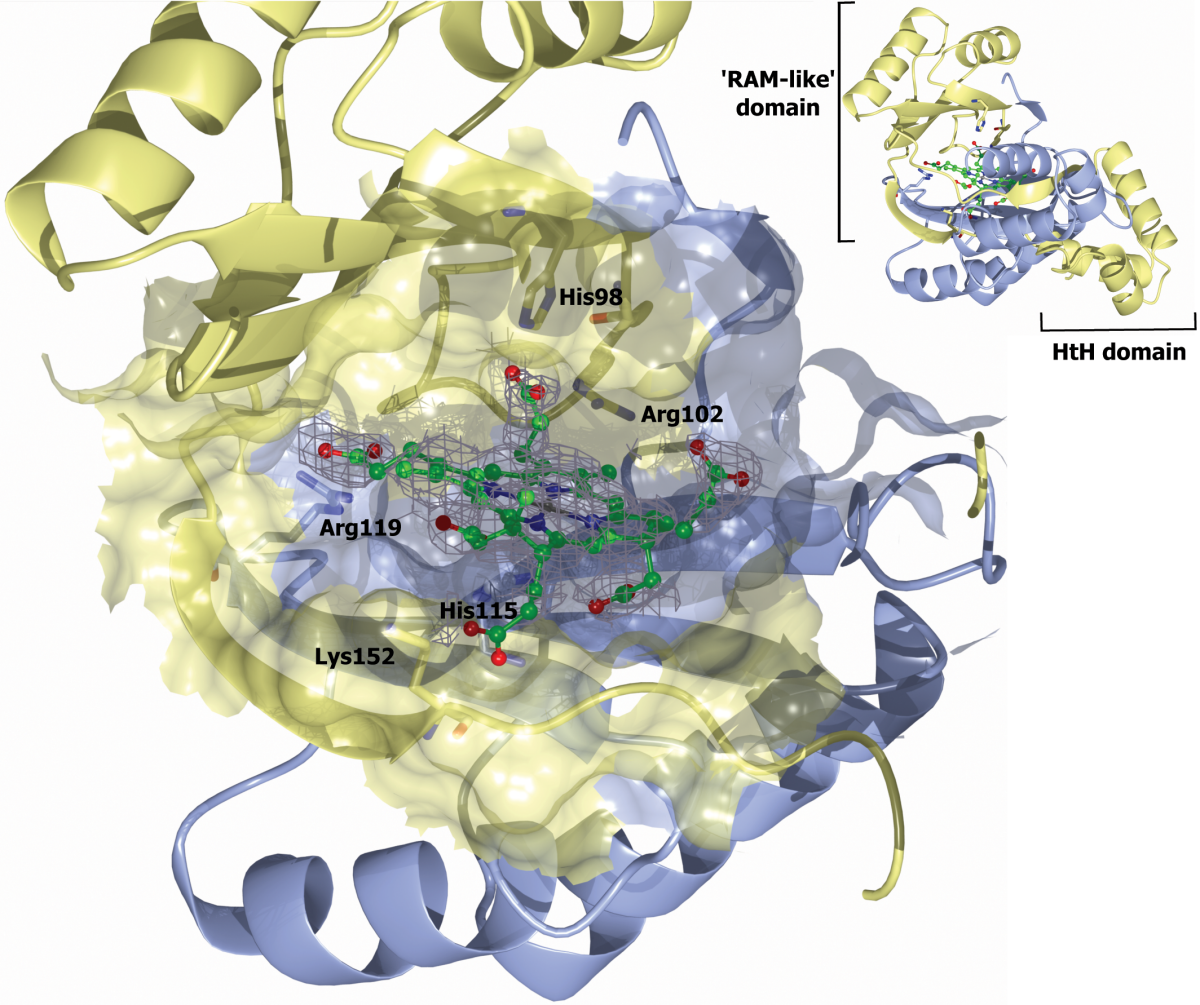
The active site of *D. desulfuricans* AhbA/B. The structure is shown with 12, 18-didecarboxysirohaem bound and interacting residues displayed as sticks. The electron density of the bound product is rendered in grey mesh, AhbA in blue and AhbB in yellow. Inset: expanded view of product bound AhbA/B complex. Coordinates and structure factor files were submitted in the protein databank with accession code 4UN1. Structural diagrams were produced using CCP4 molecular graphics program (McNicholas *et al*., 2011).

Sequence alignments of AhbA and AhbB and their homologues show a conserved HXYXR motif (Fig. S7). These conserved residues face the central cavity supporting their involvement in substrate binding and catalysis. This provides a pseudo-symmetrical binding pocket with positively charged residues suitable for binding the negatively charged tetrapyrrole substrate. To investigate this idea attempts were made at producing crystals with bound substrate, where native crystals were grown in an anaerobic chamber and soaked with sirohaem. The soaked crystals diffracted to 1.9 Å and contained additional density consistent with the presence of a tetrapyrrole. The electron density of the bound tetrapyrrole suggests the decarboxylations at C12 and C18 had occurred and the bound molecule is actually the product didecarboxysirohaem. The observation also indicates that the crystals are functional as a decarboxylase. Overall, little movement is observed upon substrate/product binding as there is only a r.m.s.d. of 0.3 Å between native and product bound complexes for the 296 equivalent Cα atoms. The structure of the complex suggests that the active site is located in the C-terminal beta sheet domain of AhbA/B. This is consistent with the results from the kinetics data, which suggest that the enzyme is functional as a heterodimer with a single active site (Fig. 8).

His115 (AhbA) coordinates the iron in the macrocycle (2.8 Å) and Arg119 (AhbA) is positioned such that it could interact with the propionate group from ring D of sirohaem (2.8 Å; Fig. 8). Lys152 (AhbB) is in a position to interact with the acetate group from ring A although the electron density for this side-chain is poor suggesting it is in disordered state in this product complex. These side-chains help to position didecarboxysirohaem with the methyl group of ring C (C12) within range of Arg102 (AhbB). We suggest this is the catalytic residue responsible for decarboxylation of this side-chain.

**Fig. 9:**
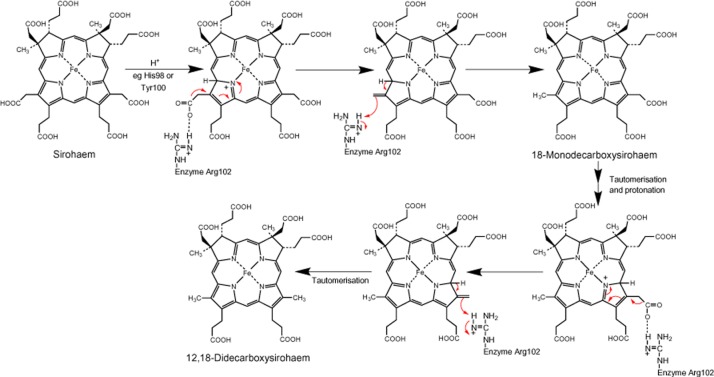
Reaction mechanism for sirohaem decarboxylase. The role of the conserved residues, His98, Tyr100 and Arg102 are highlighted.

From this arrangement we can propose a mechanism for the reaction. For convenience we will assume that the acetate side-chain on ring D is decarboxylated first. Binding at the active site has to result in a change in the double bond configuration so as to allow the electrons to run to the pyrrole nitrogen, a process that could be enhanced by Tyr100B or His98B. Stabilization of the acetic acid carboxylic acid by Arg102B would then allow decarboxylation to take place, and also provide a proton for the developing methyl group (Fig. 9). The process is then repeated for the second decarboxylation on ring C. In this respect, the product complex highlighted in Fig. 8 likely represents the events that have taken place after this second methylation. Here, ring D is proximal to Arg102B, and C11 is close to His98B. His98B is mobile in the structure, as judged from its poorly defined electron density compared with the adjacent residues, and is in a position where it can act as an acid/base, influencing double bond positioning in the pyrrole ring D and driving the reaction. The role of His98B could be the initial protonation of C11 and later its deprotonation. Tyr100B or Arg102B could act as the acid, protonating the side-chain to give the C18 methyl group. The involvement of Arg102B has been confirmed by site directed mutagenesis. Mutation of Arg102B to an alanine residue formed an inactive AhbA/B complex (Fig. S8).

For the initial decarboxylation at C18 the substrate ring will need to unbind and rebind flipped over by 180 degrees about the C5-C15 axis to bring the C18 into a similar position to that currently occupied by C12. An alternative to tetrapyrrole flipping is that the sirohaem migrates ∼ 4 Å to the second pseudo-symmetrical active centre, where there are side-chains to perform similar functions as described for the single active site model above.

From this structure it is unclear where the haem molecule would bind in the complexes from *M. barkeri* and *D. vulgaris*. As mentioned previously the proteins are active with the haem bound and given the size of the binding cleft it cannot be located in the active site with the substrate/product. Therefore further work must be undertaken to locate the haem binding site of these proteins.

## Conclusion

Despite the high degree of similarity between the AhbA and AhbB subunits from different species, it is clear there is a significant amount of cross-species variation of characteristics. All three species form heterodimeric AhbA/B complexes but only the *D. vulgaris* purifies with a clear tetrameric fraction. *D. vulgaris* and *M. barkeri* AhbA/Bs have haem binding capacity, but *M. barkeri* purifies with greater occupancy. This may be linked to the fact that haem binding affects the catalytic activity of the *M. barkeri* complex, whereas no such effect is observed with the *D. vulgaris* complex.

The structure of the *D. desulfuricans* AhbA/B complex reveals that the enzyme shares the architecture of the AsnC/Lrp family of transcription factors. It is not established, however, if AhbA/B is able to bind DNA and thereby control transcription. The AhbA/B proteins have evolved a larger cavity between the two subunits, which acts as a substrate binding site within the enzyme. A number of conserved residues line this large cleft and likely assist in catalysis. In particular, the role of Arg102B is predicted to be key in facilitating the decarboxylation of the acetic acid side-chains, akin to the role played by Arg37 in human uroporphyrinogen decarboxylase. In this respect nature has evolved two very different enzymes to enhance the decarboxylations of kinetically stable substrates by very similar mechanisms.

## Experimental procedures

### DNA manipulations

DNA manipulations were performed by standard methods. Template DNA for *ahbA* and *ahbB* genes was obtained from *D. vulgaris* Hildenborough and *D. desulfuricans* (ATCC27774). The *ahbA* and *ahbB* genes from *M. barkeri* were amplified from genomic DNA (Professor R. Thauer, Marburg). *E. coli cysG* was amplified from *E. coli* JM109 by colony PCR. The PCR reactions were performed using Faststart Taq polymerase (from *Thermus aquaticus*) according to the supplier's instructions (Roche). All constructs generated by PCR were confirmed to be correct by sequencing. Plasmids containing *cysG* and *ahbA/B* were obtained using the link and lock technique as described previously (McGoldrick *et al*., 2005; Deery *et al*., 2012), using *cysG* and *ahbA* in a pET3a vector and *ahbB* in a pET14b vector to give an N-terminal hexa-His-tag fusion to AhbB only.

### Recombinant protein production, cell growth and over expression

*E. coli* BL21 (DE3) cells were used for protein overproduction by transformation with the appropriate plasmid. Cells were grown with aeration at 37°C in LB medium containing 100 mg l^−1^ ampicillin to an OD_600_ of 0.6–0.8. Protein expression was then induced with 0.4 mM IPTG and cultures were incubated at 19°C overnight. Cells were harvested by centrifugation at 3500 *g* for 15 min at 4°C (Beckman Coulter Avanti J-301). Cell pellets from 1 l of culture were resuspended in 20 ml buffer A (20 mM Tris/HCl pH 8, 100 mM NaCl, 5 mM imidazole). Cells were disrupted by sonication (Sonics Vibra Cell). Soluble cell lysate was separated from the insoluble fraction by centrifugation at 39 000 *g* for 20 min at 4°C.

Selenomethionine enriched protein was produced using the KRX strain of *E. coli*. Two hundred and fifty millilitres of LB medium, containing 0.8% (w/v) glucose and 100 mg l^−1^ ampicillin, was inoculated with a 5 ml overnight culture of KRX *E. coli* expressing the *D. desulfuricans* pET3a *ahbA*/*ahbB* plasmid. This culture was grown overnight at 37°C (< 18 h) before being harvested and resuspended in enrichment medium. Enrichment medium contained 1× M9 media with 1% (v/v) glycerol, 1× trace elements (Bernard and Payton, 2001) and 125 mg l^−1^ selenomethionine. Cells were incubated at 25°C for 30 min to 1 h before induction with 0.2% (w/v) rhamnose for 1 day. Incorporation of selenomethionine was confirmed by mass spectrometry.

### Protein purification

All proteins were purified using nickel affinity chromatography. Cleared cell lysates were applied to a charged nickel-Sepharose column equilibrated in buffer A. The column was washed with 5 column volumes of buffer A, 5 column volumes of buffer B (20 mM Tris/HCl pH 8, 100 mM NaCl, 50 mM imidazole) and eluted in buffer C (20 mM Tris/HCl pH 8, 100 mM NaCl, 400 mM imidazole) in 2 ml fractions. The two highest protein-containing fractions were buffer exchanged into buffer D (20 mM Tris/HCl pH 8, 100 mM NaCl) using a PD-10 column (GE Healthcare Life Sciences) to remove any imidazole. Protein used for crystallography was purified in buffers containing 20 mM HEPES pH 8 instead of Tris/HCl.

### Production of sirohaem

Sirohydrochlorin was produced using the multi-enzyme approach as described previously (Schubert *et al*., 2002). A 10-fold excess of FeSO_4_ was added to the sirohydrochlorin solution and was incubated at room temperature for 4 h. Sirohaem was then purified on a DEAE column equilibrated in buffer D. Sirohaem was eluted using a gradient from 100 mM to 1 M NaCl to remove excess iron and porphyrins from the sample.

### Crystallography of *D. desulfuricans* AhbA/B

After affinity purification *D. desulfuricans* AhbA/B was subject to gel filtration on a G200 Superdex column (GE Healthcare). Protein fractions relating to a homogeneous dimer species were collected and concentrated to 11 mg ml^−1^ prior to setting up hanging drop vapour diffusion crystallization at 19°C. Initial crystals were obtained in 0.2 M ammonium acetate, 0.1 M sodium acetate pH 4.5, 30% (w/v) PEG 4000 (Molecular Dimensions, MD01 condition 2). Crystal optimization required the addition of 0.01 M BaCl_2_ (Hampton Research Additive Screen, HR2-420) and resulted in two crystal forms, short wedge shaped crystals and rectangular needle-like crystals the best diffracting being the latter. Crystals formed within 3 days. Native crystals were found to belong to the space group P2_1_2_1_2_1_ and had a Matthews coefficient predicted to have 2 copies of the AhbA/B heterodimer in the asymmetric unit. Data for native crystals was collected using beamline IO4-1 at the Diamond Light Source (UK), see Table 1 for crystallography data collection and statistics. All data was processed using the xia2 pipeline with a resolution cut-off at CC1/2 0.5.

Selenomethionine incorporated protein crystals were grown in the same conditions as the native protein and were found to belong to the same space group and similar cell dimension measurements with 2 copies of the heterodimer in the asymmetric unit. These crystals were significantly smaller and thinner than native protein, but had the same morphology. Selenium incorporation was confirmed by mass spectrometry and an initial fluorescence scan at the X-ray source. Selenomethionine data were collected using beamline IO2 at the Diamond Light Source (UK).

Selenium substructure and initial chain tracing was determined with Autosolve (Solve/Resolve) software from the PHENIX package (Adams *et al*., 2010) using multiple wavelength anomalous dispersion (MAD) data. Initial models were rebuilt in Coot (Emsley *et al*., 2010) using maps and phases calculated by Solve/Resolve to a resolution of 2.8 Å, and further validated using Buccaneer (Cowtan, 2006). Initial model building and refinement used loose NCS restraints, which were relaxed in final refinement. The native AhbA/B model was built using molecular replacement of the selenomethionine model using PhaserMR (McCoy *et al*., 2007) in the CCP4 suite (Winn *et al*., 2011) and refined using Refmac (Vagin *et al*., 2004) and manual model rebuilding. Final refinement was performed using BUSTER (Global Phasing Ltd). AhbA/B was refined at 2.23 Å to a crystallographic R-factor of 22.2% (*R*_free_ = 24.8%). Substrate soaked crystals were refined using Refmac5 (CCP4 suite) at a resolution of 1.97 Å to an R-factor of 20.5% (*R*_free_ = 23.3%).

To obtain substrate bound crystals native crystals were grown in an anaerobic chamber (< 10 ppm oxygen, Belle Technology Ltd) in the identical buffer conditions. Crystals were soaked in well solution containing 1 mM sirohaem and 10% (v/v) glycerol for 1 day. Substrate uptake was confirmed by the crystals turning purple. Data for substrate soaked crystals was collected at the European Synchrotron (ESRF; France). Coordinates and structure factor amplitudes have been deposited in the protein databank with accession codes: 4CZD (AhbA/B) and 4UN1 (AhbA/B bound to didecarboxysirohaem).

### Enzyme trapping of didecarboxysirohaem

BL21 (DE3) *E. coli* was transformed with the appropriate plasmid as described previously. Cells were cultured in 2YT media for 18 h at 28°C. Cultures were then supplemented with 0.02 g l^−1^ of 5-aminolevulinic acid and grown for a further 6 h. Proteins were purified in an anaerobic chamber to maintain the stability of the sirohaem/didecarboxysirohaem.

Proteins were purified on a nickel column and washed with 5 column volumes of buffers A and B. The column was then washed with 5 column volumes of buffer D before being washed with buffer D containing 8 M urea. This unfolded the proteins and released the bound product. The released tetrapyrrole was further purified on a TELOS C18 column (Kinesis) equilibrated in 0.1% (v/v) TFA and eluted using 100% acetonitrile before being analysed by HPLC-MS.

### High performance LC-MS analysis of sirohaem, sirohaem derivatives and haem

Tetrapyrrole intermediates were separated by acidification with equal volumes of 0.1% (v/v) TFA before purification on a TELOS C18 column, eluting in 100% acetonitrile. Samples were resolved on an ACE 5AQ column (2.1 × 150 mm, Advanced Chromatography Technologies) attached to an Agilent 1100 series HPLC equipped with diode array detector and coupled to a micrOTOF-Q (Bruker) mass spectrometer. The column was developed with a binary gradient at a flow rate of 0.2 ml min^−1^. Solvent A was 0.1% (v/v) TFA and solvent B was acetonitrile.

For sirohaem and sirohaem derivatives the column was equilibrated with 5% B. Following sample injection the concentration of B was increased to 20% over 6 min and then to 30% at 25 min and 100% at 35 min where it was held for 5 min before returning to start conditions. The total length of each run was 50 min. For samples containing haem a linear gradient was used starting at 20% B and reaching 100% B in 30 min.

### Pyridine haemochrome assay

Pyridine haemochrome assays were performed as previously described (Berry and Trumpower, 1987). Spectra were recorded between 620 and 520 nm. 0.5 ml 200 mM NaOH, 40% (v/v) pyridine and 3 μl 0.1 M potassium ferricyanide were placed in a cuvette. A 0.5 ml protein sample was added and mixed thoroughly before an oxidized spectrum was recorded. Solid sodium dithionite was added (2–5 mg) and several reduced spectra were taken. The oxidized spectrum was subtracted from the first stable reduced spectrum to provide the final pyridine haemochrome spectrum.
