# Supplementary material for: The structure, function and properties of sirohaem decarboxylase - an enzyme with structural homology to a transcription factor family that is part of the alternative haem biosynthesis pathway
Source: Mol Microbiol. 2014 Jun 18;93(2):247–61. doi: 10.1111/mmi.12656 (PMC4145669; doi:10.1111/mmi.12656)
Supplement: Supplementary file 1 — Supporting information [file mmi0093-0247-SD1.pdf]

Supporting Information for:

The structure, function and properties of sirohaem decarboxylase – an enzyme with structural homology to a transcription factor family that is part of the alternative haem biosynthesis pathway

David J. Palmer,[1] Susanne Schroeder,[1] Andrew D. Lawrence,[1] Evelyne Deery, Susana A Lobo,[2] Ligia M Saraiva,[2] Kirsty J McLean,[3] Andrew W Munro,[3] Stuart J Ferguson,[4] Richard W. Pickersgill,[5] David G. Brown[1]* and Martin J. Warren[1]*

1. School of Biosciences, University of Kent, Giles Lane, Canterbury, Kent CT2 7NJ, UK.

2. Instituto de Tecnologia Química e Biológica António Xavier, Universidade Nova de Lisboa, Av. da República, 2780-157 Oeiras, Portugal

3. Manchester Institute of Biotechnology, Faculty of Life Sciences, University of Manchester, 131 Princess Street, Manchester, M1 7DN, UK

4. Department of Biochemistry, University of Oxford, South Parks Road, Oxfordshire OX1 3QU, UK.

5. School of Biological and Chemical Sciences, Queen Mary University of London, Mile End Road, London, E1 4NS, UK

Supporting information

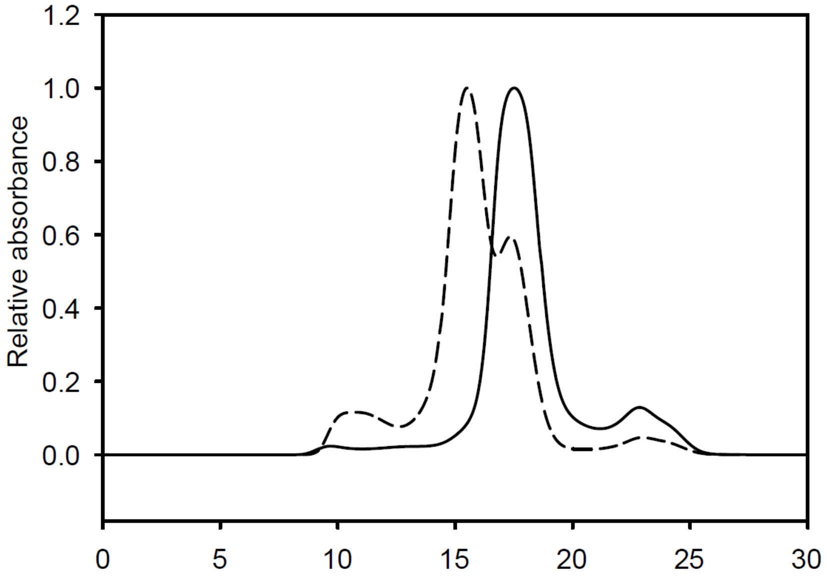

SI Figure 1. Gel filtration UV trace recorded at a wavelength of 280nm of purified AhbA/B complexes from *D. desulfuricans* (solid) and *D. vulgaris* (dashed). Major peaks correspond to a single dimeric *D. desulfuricans* AhbA/B complex and dimeric and tetrameric AhbA/B from *D. vulgaris*.

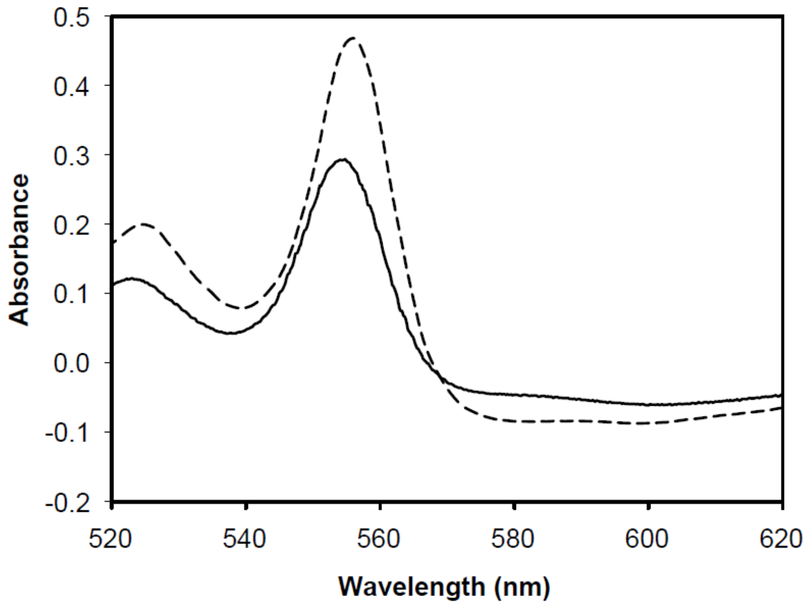

Figure S2. Difference spectra (reduced-oxidised) for pyridine hemochromes of exogenous haem-loaded *D. vulgaris* AhbAB (solid) and *M. barkeri* AhbAB (dashed). Peaks are observed at 554 nm and 556 nm, respectively.

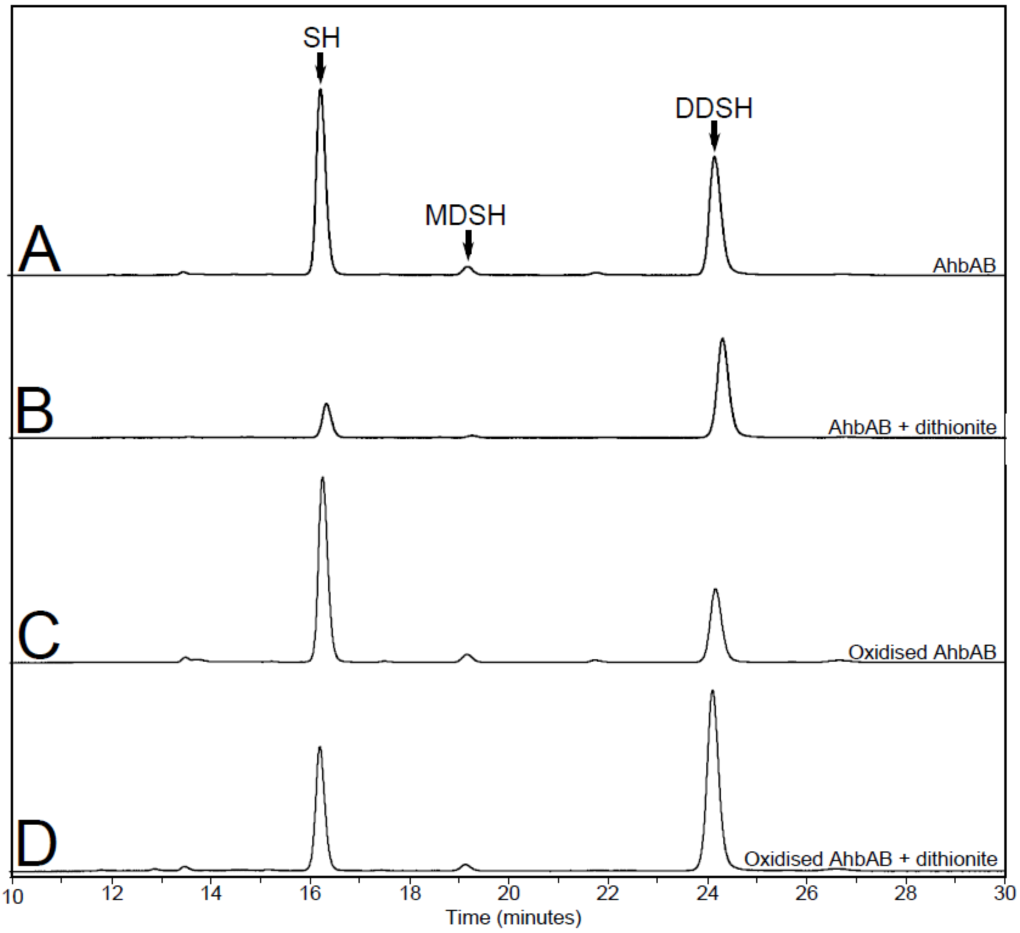

Figure S3. HPLC traces, recorded at a wavelength of 380 nm, of siroheme reactions with purified AhbA/B protein from *M. barkeri* in different oxidation states: a) purified protein, b) purified protein with a 10 fold excess of sodium dithionite, c) protein oxidised with addition of a 10 fold excess ferricyanide which was removed prior to reaction by buffer exchange, d) the same oxidised protein with a 10 fold excess of sodium dithionite. Arrows indicate peaks for siroheme (SH; ~16 min), monodecarboxysiroheme (MDSH; ~19 min) and didecarboxysiroheme (DDSH; ~24 min).

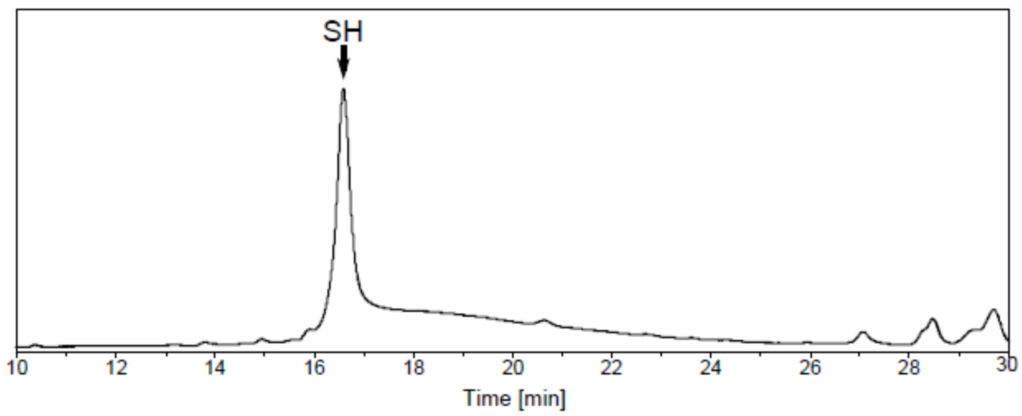

Figure S4. HPLC trace, recorded at a wavelength of 380 nm, of tetrapyrroles extracted from crude lysate of *E. coli* overexpressing *cysG*. Arrow indicates the peak for siroheme (SH; ~16 min).

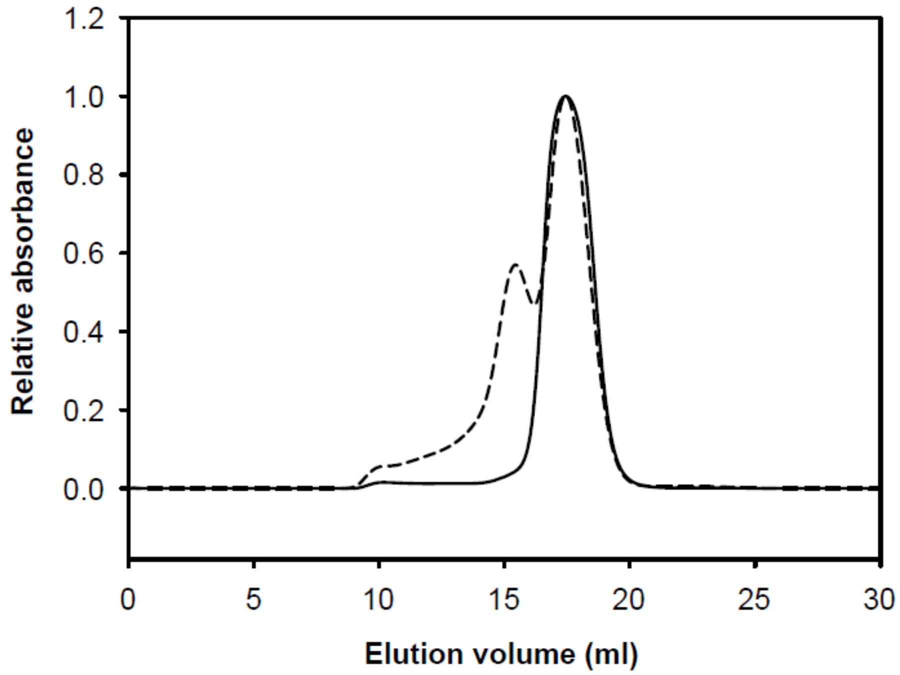

Figure S5. Gel filtration UV trace, recorded at a wavelength of 280 nm, of purified AhbA/B chimeric complexes, *D. desulfuricans* AhbA and *D. vulgaris* AhbB (solid) appears as a single heterodimeric species whereas *D. vulgaris* AhbA with *D. desulfuricans* AhbB (dashed) is present as both a dimer and tetramer.

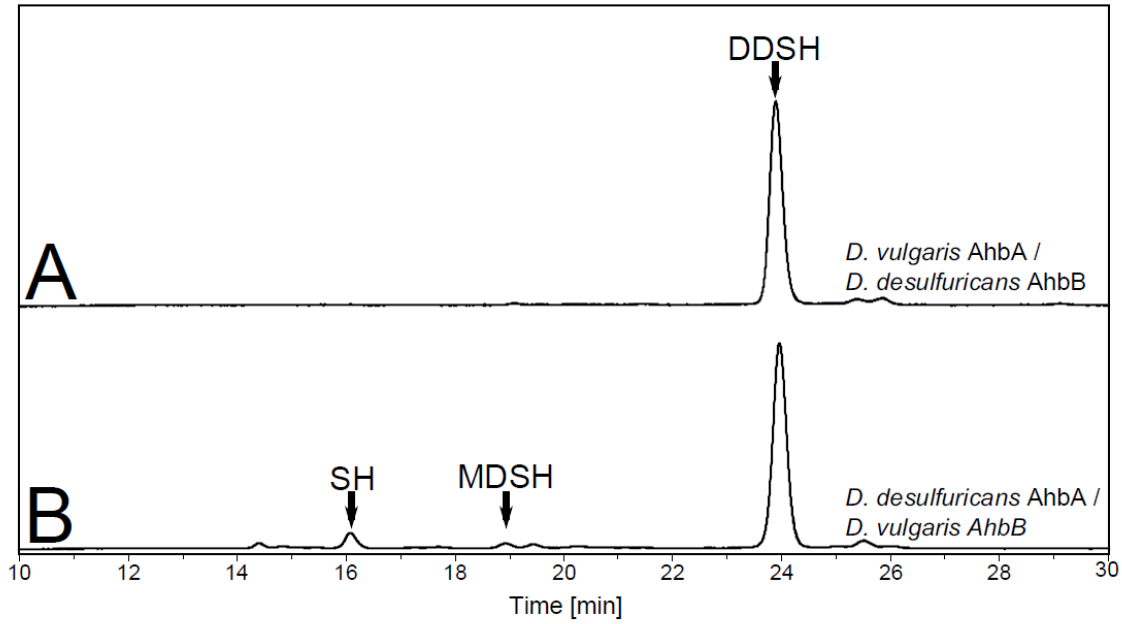

Figure S6. HPLC traces, recorded at a wavelength of 380 nm, of siroheme reactions with purified AhbA/B chimeric complexes; a) *D. vulgaris* AhbA with *D. desulfuricans* AhbB, b) *D. desulfuricans* AhbA and *D. vulgaris* AhbB. Arrows indicate peaks for siroheme (SH; ~16 min), monodecarboxysiroheme (MDSH; ~19 min) and didecarboxysiroheme (DDSH; ~24 min).

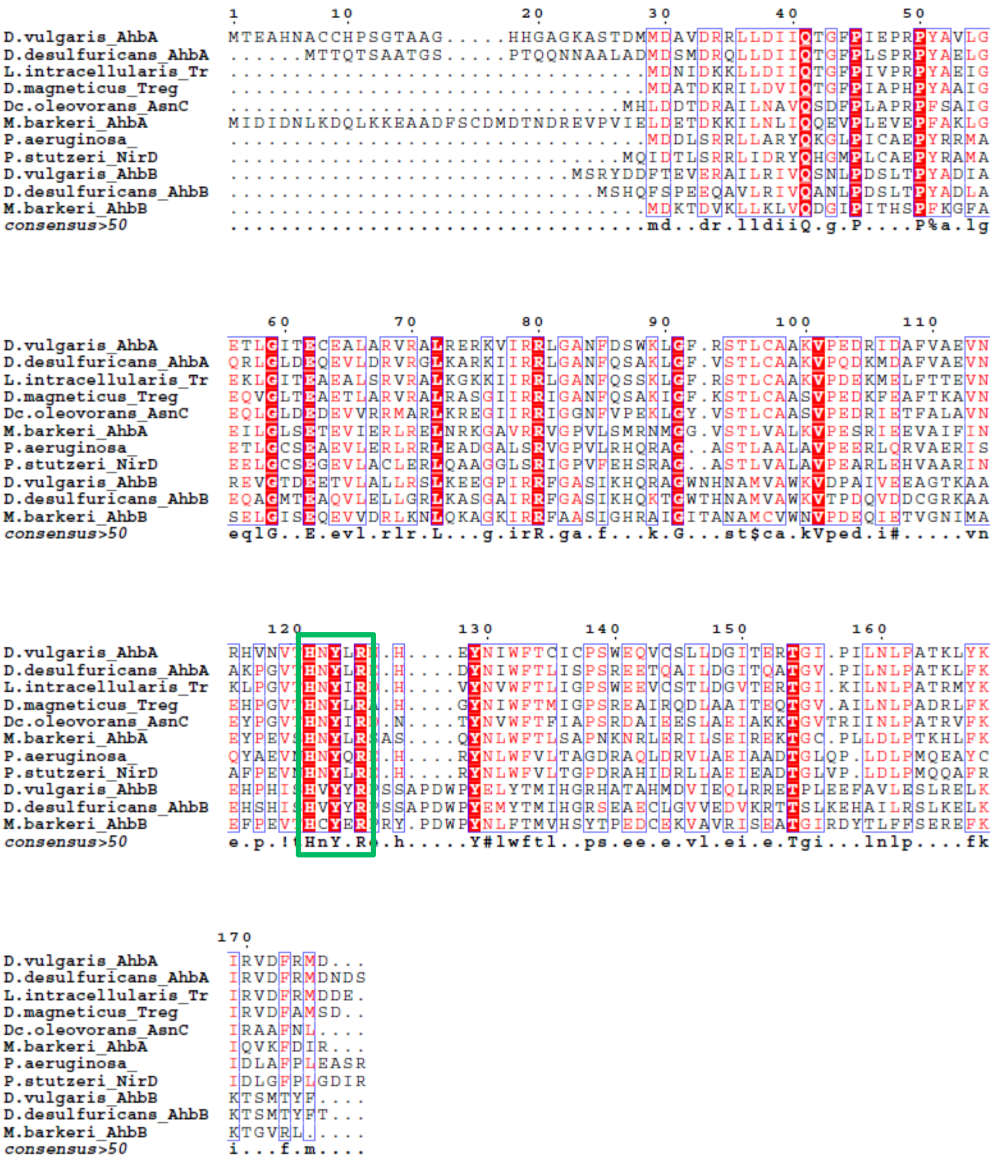

Figure S7. Sequence alignment of a number of different AhbA and AhbB sequences. Conserved residues are highlighted in red. The conserved HXYXR motif, that lines the active site of the enzyme, is boxed in green.

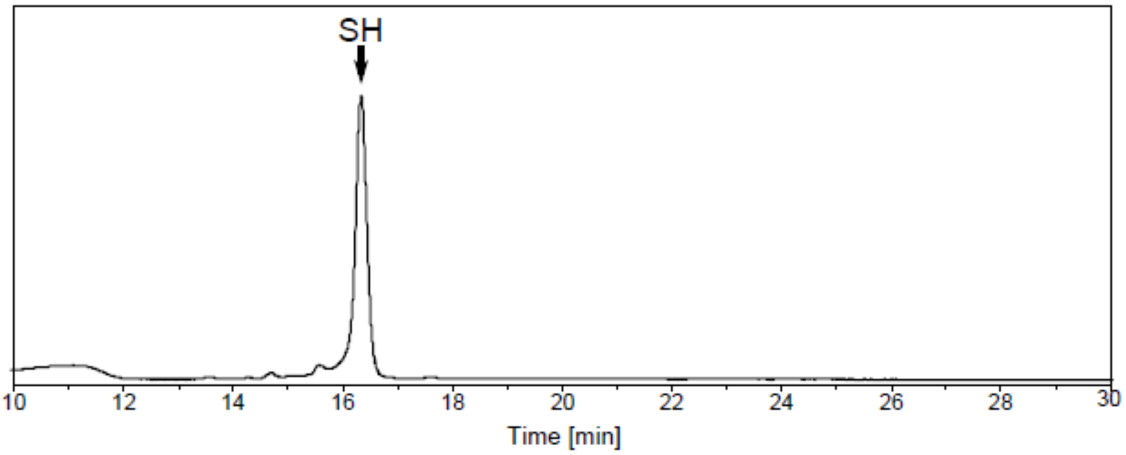

Figure S8. HPLC trace, recorded at a wavelength of 380 nm, sirohaem reaction with purified AhbA/B complex formed with AhbB mutant R102A. Arrow indicates the peak for siroheme (SH; ~16 min).
